# La replantation digitale, résultats et complications: étude d’une série de 18 cas

**DOI:** 10.11604/pamj.2016.24.184.8718

**Published:** 2016-07-01

**Authors:** Mohammed El Idrissi, Abdelhalim Elibrahimi, Mohammed Shimi, Abdelmajid Elmrini

**Affiliations:** 1 Service de Chirurgie Ostéoarticulaire B4, CHU Hassan II, Fez, Maroc

**Keywords:** Amputation, replantation, microchirurgie, Amputation, replantation, microsurgery

## Abstract

Les amputations digitales sont des lésions fréquentes, la majorité étant provoquée par des accidents de travail. Les techniques microchirurgicales représentent une alternative pour réparer ces amputations. L'objectif de ce travail est de rapporter notre expérience dans la replantation digitale à travers l'étude de 18 cas. Nous avons mené une étude rétrospective, étalée entre Juin 2013 et Janvier 2015, incluant 14 patients présentant une amputation totale ou subtotale des doigts. Nous avons inclus dans notre série toutes les replantations unidigitales et multidigitales réalisées en aval de l'insertion distale du tendon fléchisseur superficiel ainsi que les replantations digitales réalisées en amont de l'insertion distale du tendon fléchisseur superficiel. Nous avons opéré ces patients selon le procédé classique de réimplantation digitale. Cinq replantations ont été secondairement régularisées. Parmi les 18 replantations, huit replantions digitales ont favorablement évolué puisque la replantation a permis de restituer un secteur complet de mobilité passive et active du doigt opéré sans chirurgie de reprise et sans complication secondaire précoce et tardive. Dans notre étude nous avons noté des résultats satisfaisants, malgré les conditions difficiles notamment le conditionnement initial du doigt amputé, et le délai de prise en charge retardé. Le développement et la maîtrise de la microchirurgie a permis de changer le pronostic de ces amputations à retentissement fonctionnel et psychologique difficile, les résultats de notre série sont encourageants pour a mise en place d'un service SOS main au Maroc.

## Abstract (en)

Digital amputations are frequent injuries, the majority of them are caused by workplace accidents. Microsurgical techniques are an alternative option to repair these amputations. This study aims to report our experience in digital replantation through the examination of 18 cases. We conducted a retrospective study of 14 patients with total or subtotal fingers amputation between June 2013 and January 2015. All unidigital and multidigital replantations downstream of the distal insertion of superficial flexor tendon as well as all digital replantations upstream of the distal insertion of superficial flexor tendon were included in our study. These patients underwent surgery according to conventional digital replantation procedures. Five replantations were secondarily regularized. Among the 18 replantations, eight digital replantions evolved favorably since replantation helped restore active range of motion and passive range of motion of the finger operated without revision surgery and early and late secondary complication. In our study we achieved satisfactory results despite the difficult conditions including the initial state of the amputated finger and its delayed management. The development and mastery of microsurgery has helped change the prognosis of these amputations with serious functional and psychological consequences; the results of our study are encouraging with reference to the implementation of the SOS Hand Service in Morocco.

## Introduction

Les amputations digitales sont des lésions fréquentes, la majorité étant provoquée par des accidents de travail. Les techniques microchirurgicales représentent une alternative pour réparer ces amputations. La réimplantation consiste en la restauration d´une partie du corps complètement amputée [1]. La première réimplantation de pouce ayant été réalisée par Tamai et Komatsu en 1965 [1]. Le retentissement fonctionnel et esthétique des déformations provoquées par l'amputation digitale est considérable. La réimplantation, réalisée en urgence, est le seul procédé qui permette au patient de reprendre ses activités en minimisant ce double retentissement [2, 3]. L'objectif de ce travail est de rapporter notre expérience dans la replantation digitale à travers l'étude de 18 cas.

## Méthodes

**Figure 1:**
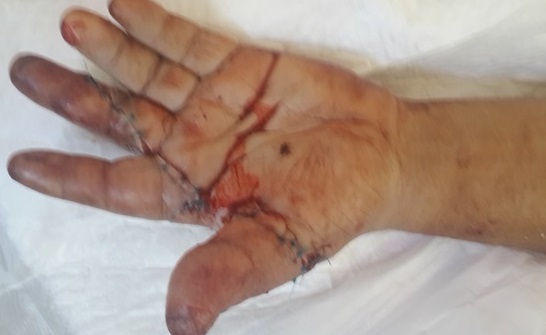
Aspect après réimplantation chez un patient présentant une amputation multidigitale

Nous avons mené une étude rétrospective, étalée entre juin 2013 et janvier 2015, incluant 14 patients présentant une amputation totale ou subtotale des doigts. Nous avons exclu de notre étude les replantations digitales réalisées dans le cadre de « ring finger » vu qu'elle nécessite une prise en charge chirurgicale particulière de ces doigts avulsés. Nous avons inclus dans notre série toutes les replantations unidigitales et multidigitales réalisées en aval de l'insertion distale du tendon fléchisseur superficiel ainsi que les replantations digitales réalisées en amont de l'insertion distale du tendon fléchisseur superficiel (trans-interphalangienne proximale et trans-première phalange). L'évaluation des résultats a porté sur la réussite ou l'échec de la revascularisation, les résultats fonctionnels. Quel que soit le niveau de la replantation digitale, la technique chirurgicale consistait, après le parage du bord des moignons en l'ostéosynthèse par des broches de Kirschner croisées. Le tendon fléchisseur (profond uniquement) est réparé par une suture de type Kessler au fil non résorbable 4/0 celle des tendons extenseurs par des points en X. Dans tous les cas, les artères digitales étaient d'abord réparées, puis les deux nerfs collatéraux et au moins deux veines dorsales. Nous n'avons en aucun cas utilisé de greffe veineuse. Une incision rétrounguéale était pratiquée dans les cas où toute anastomose veineuse était impossible. Le pansement laissait libre l'extrémité exposée de façon à ce que le segment réimplanté soit visible. Le traitement anticoagulant était composé de l'enoxaparine à une dose adaptée au poids du patient, et de l'Aspégic 1000 mg/jour. Le protocole postopératoire consistait en l'immobilisation par attelle dorsale en plâtre; membre supérieur tenu au chaud. La mobilisation des doigts en passif est recommandée à partir de la troisième semaine et la mobilisation active à partir de la sixième semaine postopératoire. La durée moyenne de l'intervention est de trois heures est demi, celle de l'hospitalisation est de 3 jours. L'attelle était habituellement laissée en place pendant six semaines. Les contrôles cliniques et radiologiques étaient systématiquement effectués à une semaine de la sortie, à trois semaines, au 3e mois, au 6e mois et à un an en postopératoire. La rééducation était habituellement prescrite pour une durée totale de six mois. Le traitement anticoagulant était habituellement prescrit pour une durée d'un mois. Selon ce protocole, nous avons opéré 14 patients, qui étaient tous de sexe masculin. L'âge moyen des patients était de 26 ans (±5). Dix d'entre eux étaient victimes d'une agression à l'arme blanche, trois d'un accident de travail et un patient victime d'un accident domestique. Dix patients avaient une amputation d'un seul doigt dont trois intéressent le pouce, et quatre patients présentaient une amputation multidigitale (Figure 1). Le mécanisme lésionnel était une section franche chez 10 patients, et un écrasement chez les quatre autres. Nous avons opéré 15 doigts longs et 3 pouces. A part quatre amputations qui siègent en aval de l'insertion du fléchisseur superficiel, le reste des patients présentaient des amputations siégeant en amont de cette insertion.

## Résultats

Cinq replantations ont été secondairement régularisées (27,7%). Trois doigts ont été régularisés pour ischémie, chez ces trois patients le siège de l'amputation était distal, l'anastomose veineuse était délicate. Les deux autres ont été régularisés suite à des troubles trophiques dont un pouce. Parmi les 18 replantations, huit replantions digitales ont favorablement évolué puisque la replantation a permis de restituer un secteur complet de mobilité passive et active du doigt opéré sans chirurgie de reprise et sans complication secondaire précoce et tardive. Par conséquent, le pourcentage de succès fonctionnel de la replantation est chiffré à 44,4%. En cas de survie de la replantation digitale, le taux de succès fonctionnel est de 61,5 % (4/13) (Tableau 1).

**Tableau 1:** Caractéristiques de la série

Patient	Age	Localisation	Mécanisme	Délai de PEC	Evolution
1	21	Index/ P3 D	Ecrasement	6 Heures	Régularisation
2	32	Index/ P1 G	Section	8 Heures	Revascularisation
2		Majeur/ P1 G	Section	5 Heures	Revascularisation
3	33	Pouce/ MP D	Section	6 Heures	Revascularisation
4	24	Auriculaire/ P1 D	Section	5 Heures	Revascularisation
4		Annulaire/ P1 D	Section	1 Heure	Revascularisation
5	35	Index / P2 G	Section	6 Heures	Revascularisation
6	26	Pouce / IP D	Ecrasement	2 Heures	Régularisation
7	18	Index/ P1 D	Ecrasement	3 Heures	Régularisation
7		Majeur/ P1 D	Ecrasement	7 Heures	Régularisation
8	28	Majeur/ IPD D	Section	3 Heures	Revascularisation
9	21	Auriculaire/ P1 G	Section	4 Heures	Revascularisation
9		Annulaire/ P1 G	Section	5 Heures	Revascularisation
10	29	Index/ P2 D	Section	1 Heure	Revascularisation
11	20	Pouce/ MP G	Section	2 Heures	Revascularisation
12	31	Annulaire/ P2 G	Section	3 Heures	Revascularisation
13	34	Majeur/ P3 G	Section	4 Heures	Régularisation
14	19	Index/ P1 D	Section	3 Heures	Revascularisation

P: Phalange, 1, 2, 3: première, deuxième, troisième, MP: Métacarpophalangienne, IPD: Interphalangienne distale, PEC: Prise en charge, D: Droit, G: Gauche

## Discussion

La possibilité de réimplanter un doigt amputé a fasciné les chirurgiens depuis longtemps [4]. La première réimplantation digitale réalisée avec succès fut celle d'un pouce [1]. Depuis lors, la technique de réimplantation microchirurgicale a subi de progressifs raffinements élargissant le champ de ses applications [5]. Bien que la viabilité des tissus soit l'élément initial à prendre en considération, elle n'est pas le seul déterminant du succès de la réimplantation. Beaucoup d'autres éléments sont à prendre en compte; en particulier le niveau et le type de lésion, le temps d´ischémie, les chances de survie, le résultat fonctionnel espéré, les caractéristiques du patient, la comorbidité, la durée de rééducation, et le coût global engagé par le patient [6]. Selon Barbary [7] les indications absolues pour la réimplantation sont: 1) les enfants chez qui la replantation doit être tentée quel que soit le niveau d'amputation car ils ont un immense potentiel d´adaptation, 2) le pouce puisque l´opposition est fondamental pour la préhension, 3) les amputations distales car elles donnent de bons résultats, par opposition aux amputations au niveau de l'interphalangienne proximale, 4) les patients avec une main mutilée où chaque doigt restant est précieux et 5) les patients avec des exigences professionnelles particulières tel que les musiciens. Les contre-indications à la réimplantation comprennent la contamination des moignons d'amputation, fractures comminutive avec importante perte de substance osseuse et les fractures articulaires avec dégâts articulaires importants [8]. La régularisation doit être également indiquée devant la présence de comorbidités associées qui entrave le résultat de la microchirurgie, le mauvais état du segment amputé, un ring finger avec avulsion des deux tendons fléchisseurs, amputation de l´index lorsqu'elle siège près de l'interphalangienne proximale car la main fonctionne mieux sans un index qu´avec un doigt raide, douloureux et souvent exclu [7]. Braga-Silva [9] rapporte les résultats de 85 replantation effectuées en ambulatoire, avec uniquement 12 échecs, sans prendre en considération le résultat fonctionnel. Dans sa série de 46 replantations, Dos Remédios [10] rapporte un taux d'échec de 37%, avec des résultats fonctionnel satisfaisant pour un cas de replantation sur cinq. Ces résultats sont meilleurs pour les replantations après section digitale par rapport aux écrasements. Mais le principal facteur déterminant dans la survie après replantation digital serait la réalisation et la réussite de l'anastomose veineuse [11]. Si l'objectif final de la replantation digitale ne doit pas se limiter à une restitution anatomique de doigts dévascularisés car le pronostic à moyen et long terme est la fonction du doigt replanté, l'information du patient et l'obtention de son consentement éclairé pour une éventuelle indication de replantation digitale s'avère primordiale [10]. Dans notre étude nous avons noté des résultats satisfaisants, malgré les conditions difficiles notamment le conditionnement initial du doigt amputé, et le délai de prise en charge retardé.

## Conclusion

Le développement et la maîtrise de la microchirurgie a permis de changer le pronostic de ces amputations à retentissement fonctionnel et psychologique difficile, les résultats de notre série sont encourageants pour a mise en place d'un service SOS main au Maroc.

### Etat des connaissances actuelle sur le sujet

- Les technique microchirurgicales sont de plus en plus maîtrisées en matière des replantations digitales, leurs résultats sont toujours satisfaisants lorsque les indications sont raisonnablement posées et que la technique est respectée.

### Contribution de notre étude à la connaissance

- Dans les pays en voie de développement ces techniques de microchirurgie restent moins vulgarisées à défaut de moyen. Les résultats de notre série nous encouragent à instaurer un service SOS main dans notre formation hospitalière.
